# The pathological and molecular but not clinical phenotypes are maintained after second passage of experimental atypical bovine spongiform encephalopathy in cattle

**DOI:** 10.1186/s12917-014-0243-2

**Published:** 2014-10-02

**Authors:** Timm Konold, Laura J Phelan, Derek Clifford, Melanie J Chaplin, Saira Cawthraw, Michael J Stack, Marion M Simmons

**Affiliations:** Animal Sciences Unit, Animal Health and Veterinary Laboratories Agency, New Haw, Addlestone KT15 3NB UK; Pathology Department, Animal Health and Veterinary Laboratories Agency, New Haw, Addlestone KT15 3NB UK; Prion Unit, Virology Department, Animal Health and Veterinary Laboratories Agency, New Haw, Addlestone KT15 3NB UK; Central Sequencing Unit, Animal Health and Veterinary Laboratories Agency, New Haw, Addlestone KT15 3NB UK

## Abstract

**Background:**

Atypical bovine spongiform encephalopathies (BSEs), classified as H-type and L-type BSE based on the Western immunoblot profiles, are naturally occurring diseases in cattle, which are phenotypically different to classical BSE. Transmission studies in cattle using the intracerebral route resulted in disease where the phenotypes were maintained irrespective of BSE type but clinically affected cattle with a shorter survival time displayed a nervous form whereas cattle with a longer survival time displayed a dull form. A second transmission study is reported here where four cattle were intracerebrally inoculated with brain tissue from experimentally infected cattle presenting with either the nervous or dull form of H- or L-type BSE to determine whether the phenotype is maintained.

**Results:**

The four inoculated cattle were culled at 16.5-19.5 months post inoculation after presenting with difficulty getting up, a positive scratch response (all) and dullness (three cattle), which was not observed in two non-inoculated control cattle, each housed with either group of inoculated cattle. Only the inoculated cattle had detectable prion protein in the brain based on immunohistochemical examination, and the Western immunoblot profile was consistent with the H-type or L-type BSE of the respective donor cattle.

**Conclusions:**

Second passage of H-type and L-type BSE in cattle produced a TSE where the majority of cattle displayed the dull form regardless of clinical disease form of the donor cattle. The pathological and molecular phenotypes of H- and L-type BSE were maintained.

**Electronic supplementary material:**

The online version of this article (doi:10.1186/s12917-014-0243-2) contains supplementary material, which is available to authorized users.

## Background

Atypical bovine spongiform encephalopathy (BSE) is a prion disease of usually older cattle that is distinct from classical BSE by its pathological, molecular and biological phenotype. Two types are recognised, H-type BSE [1] and L-type BSE (also called bovine amyloidotic spongiform encephalopathy (BASE) [2]), which can be differentiated based on distribution and type of disease-associated prion protein (PrP^d^) in the brain [2-5] as well as proteinase K (PK) digestion characteristics and migration pattern of the proteinase-resistant fragment of the prion protein (PrP^res^) on a Western immunoblot [6,7]. Atypical BSE occurs world-wide, even in those countries with a low risk of classical BSE, with a frequency of approximately 1–2 per million cattle over 8 years old, which led to the hypothesis that these types represent sporadic transmissible spongiform encephalopathies (TSEs) [8]. Furthermore, it has been suggested that atypical BSE may be the origin of the classical BSE epidemic [9], which is supported by studies in mice that showed that a disease phenotype indistinguishable from classical BSE can be induced [10-13].

However, this has not yet been reproduced in the actual host: the atypical BSE disease phenotype is maintained according to several transmission studies of naturally occurring atypical BSE to cattle by the intracerebral route [4,14-17], and this also applies to second passages in cattle using L-type-like BSE in the only study published to date [18].

Clinically, the experimental disease in cattle is characterised by locomotor changes and changes in behaviour and mental status described as either increased nervousness and anxiety or dullness, regardless of atypical BSE type. Affected cattle eventually have difficulty rising, which may explain why naturally occurring atypical BSE cases detected through active surveillance of fallen stock often have a history of dysstasia or recumbency [3,19-22].

We previously reported a transmission study of atypical BSE in cattle to generate tissue for reference and European quality assurance, which produced pathological and molecular phenotypes consistent with the original source (H-type and L-type BSE) but it produced two clinical phenotypes: a nervous form, and a dull form only seen in cattle with the longest survival time [4]. This report describes the findings in a subsequent tissue generation study, which provided the opportunity to determine whether the clinical, pathological and molecular phenotypes are maintained upon subpassage in cattle by inoculating cattle with brains from experimental H-type and L-type BSE-affected cattle displaying either the nervous form or dull form.

## Methods

All procedures involving animals were approved by the Home Office of the UK government according to the Animal (Scientific Procedures) Act 1986 under project licence 70/7312.

### Inocula

Brain homogenates (cerebral cortex in 10% w/v solution in sterile saline) were prepared from four cattle intracerebrally inoculated with H-type BSE (N = 2) and L-type BSE (N = 2) brain in a study reported previously [4]. The donor cattle were selected on the basis of their clinical presentation and survival time (nervous form – shorter survival time, dull form – longer survival time) as follows:

H-type BSE: steer H1 (nervous form, survival time 17 months) and steer H4 (dull form, survival time 21.5 months).

L-type BSE: steer L1 (nervous form, survival time 17.5 months) and steer L4 (dull form, survival time 23 months); for detailed information on donor cattle see previous report [4].

All inocula were tested for bacterial contamination and treated with antibiotics if required (H4 and L1 brain homogenate) according to established methods [23].

### Animals

Four Hereford crossbred calves sourced from the UK were intracerebrally inoculated at 7 months of age with 1.0 ml of either L-type or H-type BSE brain homogenate according to previous published methods ([23]): steer H5 and heifer H6 with inoculum from donors H1 and H4 respectively, and steer L5 and heifer L6 with inoculum from donors L1 and L4 respectively. EDTA blood was collected from each animal for DNA extraction to determine the PrP gene open reading frame (ORF) polymorphisms and promoter region as described previously [24]. Both groups each included a third, age-matched calf as an environmental control, which was not inoculated: Aberdeen Angus crossbred heifer CO3 for the H-type BSE inoculated cattle and Holstein Friesian steer CO4 for the L-type BSE inoculated cattle.

Selection of cattle groups was purely based on availability at the start of the study, given that there has been no evidence that the pathological or molecular phenotype varies between breeds or gender (Hereford crossbred steers or heifers as recipients versus Aberdeen Angus crossbred steers as donors), but it was decided to use the same breed for the inoculated cattle to enable comparison between phenotypes.

### Husbandry and routine procedures

Both groups were housed in medium security accommodation with no contact between groups, with separate pen entrances and separate equipment. All housed cattle were given hay *ad libitum* and a daily concentrate ration free from meat and bone meal. Routine procedures included monthly weighing of all cattle, one pre-inoculation and then monthly blood sampling from 1 month post inoculation (mpi) of inoculated cattle until 13 mpi when routine blood sampling was discontinued to prevent stress during sampling and enable comparison of the clinical presentation with the control cattle, which were not sampled.

### Clinical assessments

All animals were clinically examined prior to inoculation to confirm their suitability for the study.

#### Behavioural observations

Animals were checked daily by animal husbandry staff and any unusual behaviour during feeding or cleaning of the pens was recorded. Scientific or veterinary staff carried out weekly observations for 15 minutes per pen between 10:30 a.m. and 12 noon to assess the animals’ behaviour and mental status and reactivity [23]. These observations, which started from 9 mpi were conducted without interaction of the observer except for the ‘hand approach test’ (moving the hand towards the animal when close to the observer at the door) or the clipboard test (waving a clipboard towards the animal) at the end of the observation period if an animal did not approach.

Similar to the previous study, animals were also monitored during daytime by closed circuit television using two cameras (AXIS 209FD with Camera Station software version 3, AXIS Communications, Lund, Sweden) mounted in opposite corners of each pen. Weekly camera observations were conducted from 10.5 mpi to assess the cattle’s rising behaviour and general activity. Rising behaviour was later also specifically assessed by animal husbandry staff when the first difficulty in getting up was noticed.

The scoring system for rising behaviour was similar to that used in the previous study:

score 0 = getting up without delay, rising on hind limbs first;

score 1 = slight difficulties getting up, e.g. the phase of rising on the hind limbs seems to be delayed;

score 2 = obvious difficulties getting up with rising on the fore limbs first or dragging the body along the floor before getting up;

score 3 = unable to get up, the animal may attempt to do so but does not succeed; this score was also given to cattle that were unable to get up but got up when aided by staff.

### Neurological examinations

Neurological examinations were conducted at 6, 9 and 12 mpi and monthly thereafter. As in previous studies, clinical examinations consisted of a neurological examination according to a standard protocol [23], which included inspection of the retina and tests of over-reactivity, such as testing the response to camera flash (‘flash test’), to hand clapping and a metallic bang, to a clipboard waved in front of the animal (‘clipboard test’) and to touching of the hind limbs with a flexible stick (’stick test’) [25]. In addition, testing of the scratch response was carried out from 17 mpi whereby the back of cattle was scratched to assess whether a repeatable, stereotypical response could be elicited. This test had been used routinely in sheep with TSE, with nibbling movements of the lips, lip licking (“nibble reflex”) or rhythmical head or body movements being scored as positive [26]. All animals were assessed by the same veterinary clinician but due to the breed differences it was known to the veterinary and scientific staff from the outset which animals were inoculated with atypical BSE although the BSE-type (H- or L-type) was not disclosed.

### Clinical end-point

Cattle were culled by intravenous injection of pentobarbitone at clinical end-point when they displayed signs in at least two of the three categories ‘changes in mental status, behaviour and activity’, ‘changes in sensation’ and ‘changes in posture and movement’ or – based on previous experience with the clinical picture of experimental atypical BSE in cattle where behavioural or gait changes may be more subtle than in classical BSE [4] – had considerable difficulty getting up likely to result in permanent recumbency. Control cattle were culled when the last inoculated animal in their pen was culled.

### Postmortem diagnostic tests

#### Histopathology and immunohistochemistry

The brain of each inoculated animal was removed and the right half fixed in 10% formal saline for histopathological and immunohistochemical examination, and the left half was frozen at −80°C. Sections representing standardised levels of the fixed brain [27,28] were prepared from paraffin wax-embedded blocks using routine histological methods and stained with haematoxylin and eosin (H&E) for the detection of vacuolar changes as described previously [27]. Further sections were immunolabelled with the rat anti-PrP monoclonal antibody (mAb) R145 (Animal Health and Veterinary Laboratories Agency, Addlestone, UK) for the detection of PrP^d^ as described in detail elsewhere [28,29].

Immunohistochemical examination was also performed on spinal cord sampled at four levels (C_6–7_, T_4–5,_ T_9–10_ and L_2–3_), mesenteric lymph node, distal ileum, palatine tonsil, medial retropharyngeal lymph node, trigeminal ganglion and extraocular muscles, together with samples from the triceps, medial gluteal and semitendinosus muscles from all animals except L6, from which only limited samples could be taken.

Only the brainstem was sampled from control cattle for histopathological and immunohistochemical examination.

### Western immunoblotting

Fresh, frozen samples of brainstem from inoculated cattle were processed using the BioRad TeSeE universal Western blot (WB) kit (BioRad Laboratories, Marnes-La-Coquette, France) according to the manufacturer’s instructions as described previously [4]. The mAbs used to detect the proteinase-resistant fraction of the prion protein (PrP^res^) were SHA31 (BioRad Laboratories, included in the WB kit), P4 (R-BioPharm, Darmstadt, Germany) and SAF84 (Cayman Chemicals, Ann Arbor, USA). Where necessary, sample dilutions were carried out in Laemmli sample buffer (BioRad Laboratories) prior to loading on the gel. The signal was visualised by chemoluminescence and the image captured using a Fluor-S multiImager (BioRad Laboratories).

## Results

### Animal data and clinical disease

Genotyping revealed that all inoculated cattle had PrP alleles with six octapeptide repeats (6:6); the cattle inoculated with H-type BSE also had silent single nucleotide polymorphisms at codon position N192 (AAY instead of AAC) within the PrP gene ORF. All animals were homozygous for the deletion in the 23 bp and 12 bp indels.

**Figure 1:**
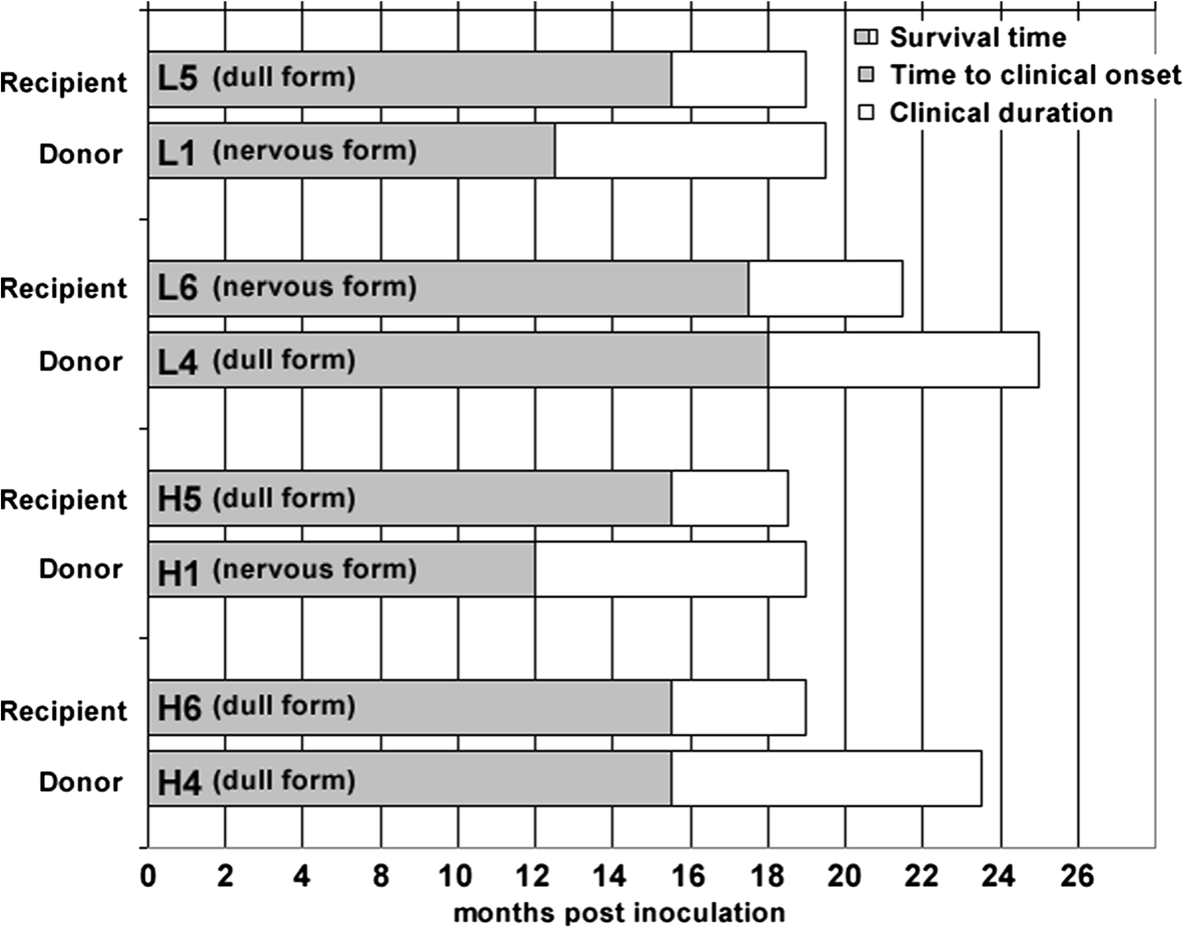
**Time to clinical onset, duration and survival time in atypical BSE brain donors and recipients.** Comparison of clinical onset, duration and survival times between H- and L-type BSE brain recipients L5, L6, H5 and H6 and the respective H- and L-type BSE donors L1, L4, H1 and H4, which were intracerebrally inoculated with atypical BSE brain from naturally occurring cases. The survival times of both H-type recipients H5 and H6 and L type recipient L5 were similar, with only 20 days between the first (H5) and the last (L5 and H6) culled animals, whereas animal L6 was culled two months after animals L5 and H6. Clinical duration and survival times were longer in the atypical BSE brain donors, whereas clinical onset was earlier in two of the atypical BSE donors, L1 and H1, compared to the recipients L5 and H5 respectively.

All inoculated cattle developed progressive neurological signs suggestive of BSE. The estimated clinical onset, characterised by the display of subtle gait changes with difficulty getting up or behavioural abnormalities, was between 13.5 and 16.5 mpi and the clinical duration was 3–4 months. Control cattle were culled at 17 months (CO3 in H-type group) and 19.5 months (CO4 in L-type group) after test group inoculation. Figure 1 shows times of clinical onset, clinical duration and survival time (in mpi) at clinical end-stage for each inoculated animal and its corresponding donor of the atypical BSE brains.

**Figure 2:**
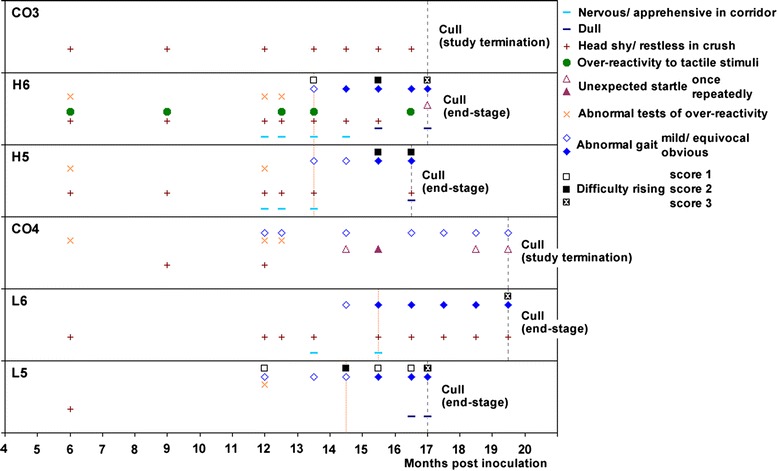
**Clinical findings in L- and H-type BSE cases over time compared with controls.** Difficulty getting up and gait abnormalities were seen in all four cases, dullness in three cases. Clinical findings were derived from observations and examinations. The horizontal axis represents the time points when neurological examinations were done; a symbol at a particular time point indicates that the sign was observed between this and the previous time point. The vertical, dotted orange lines indicate estimated clinical onset, the vertical, grey interrupted lines the time of cull. Head shy/restless in crush: e.g. head tossing or backing off when faced in crush. Over-reactive to tactile stimuli: e.g. head tossing in response to touching of the head. Abnormal tests of over-reactivity: startle at flash, clipboard test or hand clap, kicking on stick test. Abnormal gait: e.g. dysmetria.

Selected clinical signs observed in the inoculated cattle over time and clinical findings in control cattle for comparison are displayed in Figure 2.

The predominant clinical sign in all cattle was difficulty getting up, characterised by rocking several times before getting up (score 1), crawling along the floor before getting up (score 2, see additional file 1 – H5 where the animal can be seen rocking and briefly crawling along the floor before getting up) and inability to get up (score 3, not seen in animal H5), even though the animals were able to rise without major difficulty when aided. At the first detection of difficulty getting up, cattle had usually displayed mild hind limb gait abnormalities (hind limb ataxia and hypermetria), which became more obvious as the disease progressed. Behavioural and sensory abnormalities (apprehension in the corridor, restless behaviour when restrained in a crush, over-reactivity to external stimuli or unexpected startle reactions) were seen from the first examination after inoculation but did not progress and were also displayed in control cattle. With the exception of L6, all other inoculated cattle (H5, H6 and L5) became dull, which was most evident on passive observations and CCTV observations: affected cattle stood with their heads close to the wall or dividing gate or occasionally rested their heads against them. Whilst the eyes were closed, the cattle then gradually lowered their heads towards the floor (see additional file 1 – H5, which shows this steer gradually lowering its head along the wall).

Unexpectedly, all inoculated cattle displayed a positive scratch test, characterised by lip movements when the tail head was scratched. Scratch testing was not routinely performed in cattle, and a positive response was first noticed by coincidence in animal H5 prior to cull when the animal was scratched on the tail head in the pen (see additional file 1 – H5, which shows the steer moving its lips in response to scratching the tail head). The scratch test was subsequently done in the other inoculated cattle, which responded similarly, whereas no response was elicited when the tail head of the control cattle was scratched. Over the course of two weeks, the regions that elicited a positive scratch response extended further rostral in animal L6, which had the longest survival time (see additional file 2 – L6, which shows the progression in the response to the scratch test). None of the cattle exhibited any skin condition or lesion at the area that elicited a scratch response.

### Postmortem test findings

#### Histopathological and immunohistochemical examination

None of the control cattle had detectable vacuolar changes or PrP^d^ immunolabelling in the obex.

All four challenged animals displayed vacuolar lesions and immunopathology, which was completely consistent with the morphology and neuroanatomical distribution of immunolabelling previously described in the donor animals [4]. Briefly, extensive particulate and small aggregate immunolabelling was present throughout the neuraxis of all four animals, with glial labelling a predominant additional feature in H5 and H6 and perineuronal labelling in L5 and L6. Both patterns of immunolabelling were distinct from each other, and different from classical BSE. As before, there was no observed involvement of the lymphoreticular system in any animal, and peripheral immunolabelling was confined to muscle spindles. There was no evidence of amyotrophic lesions in examined sections of the gluteal and triceps muscles.

### Western immunoblot examination

**Figure 3:**
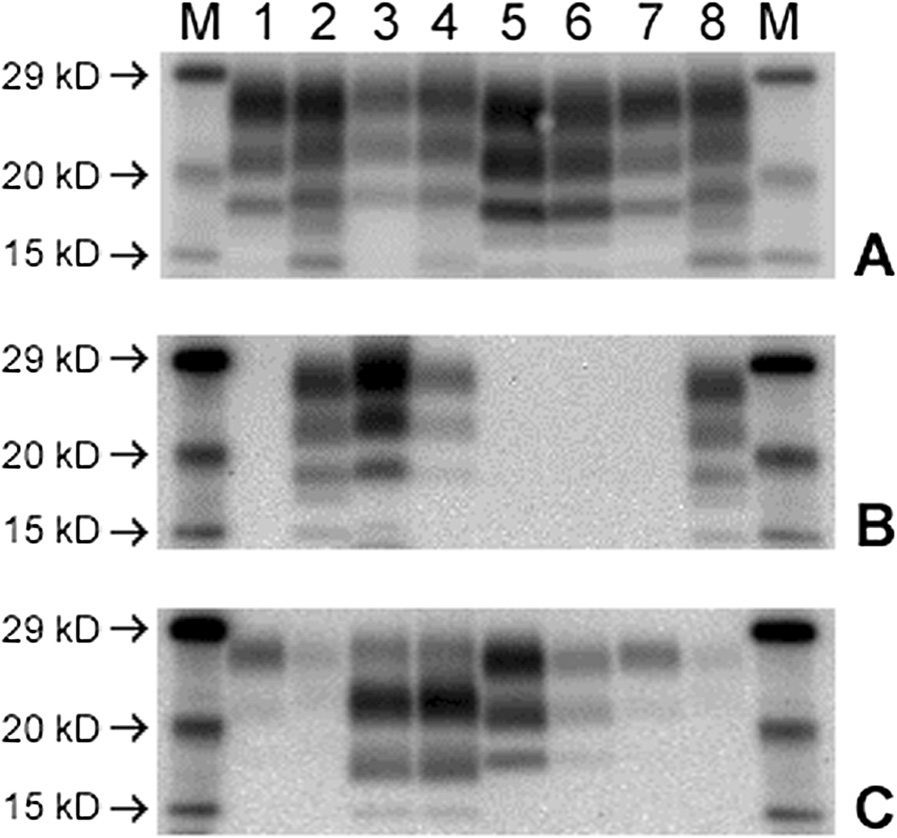
**Western immunoblot of brainstem samples from the four recipients detected with three different antibodies. A**: mAb SHA31, **B**: mAb P4 and **C**: mAb SAF84. Lane Key: M Molecular mass marker. 1 & 7 Classical BSE control. 2 & 8 Classical scrapie control. 3 H5. 4 H6. 5 L5. 6 L6.

The WB profile displayed by each of the four recipients conformed to the recognised characteristics of H or L-type BSE when detected with each of the three mAbs. Furthermore there was no apparent difference in the profiles between the nervous and dull clinical types (see Figure 3).

Essentially, H-type BSE displayed a high molecular mass migration with mAb SHA31, detection at a similar intensity with mAb P4 and a reduced volume of PrP^res^ in the diglycosylated band and additional low molecular mass band at approximately 14 kD with mAb SAF84.

L-type BSE displayed a low molecular mass migration with both mAbs SHA31 and SAF 84, similar ratio of PrP^res^ in the di- and monoglycosylated bands with mAb SHA31 and was not detected with mAb P4.

## Discussion

H-type and L-type BSE are prion diseases of cattle with distinct differences in the pathological and molecular phenotype, which are maintained after experimental transmission of naturally occurring disease to cattle [4,5,15]. Both the PrP^d^ immunolabelling pattern and the visual WB PrP^res^ profiles observed in the secondary passage recipient cattle maintained the same characteristics as the respective donor and primary passage recipients previously published [4], which confirmed that no change in the pathological and molecular H- or L-type profile occurred upon subpassage in the bovine host. This had been previously described for the Japanese BASE-like isolate BSE/JP24 [18]. Thus, the current study did not provide any evidence that the atypical BSE characteristics may change into classical BSE following subpassage in cattle. Transmission studies in wild-type mice have shown that both atypical BSE types may convert into classical BSE [10,12,30], and this was also demonstrated in bovine transgenic mice for H-type BSE [11]. If this phenotype change, which is seen only inconsistently in mice, also occurs in cattle, it may not be detectable in the few studies of atypical BSE in cattle, which generally have only small sample sizes.

The current study showed that difficulty getting up remained the major clinical sign in the H- and L-type BSE-affected cattle, which is consistent with the previous [4] and other studies conducted elsewhere [14-18]. Muscle atrophy and fasciculations as observed in experimentally infected BASE-affected cattle [14] were not observed. Although all affected cattle were able to get up unaided from a sternal lying position even at clinical end-stage, three cattle were found in a lateral position and unable to right themselves unaided, which would have ultimately resulted in death. If naturally occurring atypical BSE has a similar clinical presentation, it is unlikely that these cases would have been associated with BSE under normal farming practice and reported as suspects unless the recumbency had been preceded by gait abnormalities that had been recognised by the herdsman. This supports the field situation, in which the great majority of atypical BSE cases have been identified through the statutory active screening of fallen stock and emergency slaughter populations [31]. In the absence of reported clinical suspects, detailed description of the clinical phenotype to aid in the recognition of clinical disease in the field can only be obtained from experimental studies.

Signs often observed in classical BSE, such as over-reactivity to external stimuli, unexpected startle responses, apprehension and anxiety, which were also present in the atypical BSE-affected donor cattle and interpreted as the ‘nervous disease form’ [4], were not a feature in the current study, although head tossing, nose licking and teeth grinding associated with classical BSE [32] were seen in one animal (H5). This steer and two other cattle became dull, including L5, whose donor had never displayed the dull form of the disease. Surprisingly, the animal with the longest survival time and longest clinical duration (L6) did not appear to be dull (classified as displaying the nervous form in Figure 1 even though head shyness was the only sign of over-reactivity), whereas the donor animal had displayed the dull form. Although based on only one animal, it does not support the hypothesis that affected animals become duller as the disease progresses, even though lesions in the brain become more widespread and may affect the forebrain, diencephalon and the reticular activating system in the brainstem, which are associated with an altered state of alertness [33]. The lack of a clear relationship between morphological changes or PrP^d^ accumulation and clinical signs has been reported before in large animals affected by prion diseases [34-36]. There were no obvious differences in the neuropathology of individual cattle that could explain the difference in the clinical presentation and there was also no discernable molecular profile difference between the two L-type recipients (presence or absence of dullness).

Dullness was consistently reported as a sign of H- or L-type BSE-affected cattle in other studies [14-17]. We cannot exclude the possibility that the display of nervousness is dependent on breed, as it has been shown for classical BSE where the occurrence of temperament changes and kicking was less frequent in Guernsey cattle [37]. As reported for the previous study, the prion protein genotype did not appear to affect the clinical presentation as both dull and non-dull forms occurred in cattle with the same genotype, inoculated with L-type BSE.

Similarly, a longer survival time in the donor was not necessarily maintained in the recipient because the survival times in the H-type affected cattle were similar whereas there had been a difference of more than 4 months in the donors. The clinical duration appeared to be much more uniform and similar in all recipient cattle, even though the inoculum was derived from different cattle, whereas the duration in the donors had been more variable despite the use of the same inoculum for H-type and L-type inoculated cattle respectively. This is most likely due to the reliance on certain clinical markers to define clinical onset, which were difficulty getting up and gait abnormalities in the absence of behavioural abnormalities that were clearly distinguishable from control cattle in the present study, whereas the donor cattle displayed over-reactivity relatively early in the incubation period, which was associated with disease, because it was not seen in the control cattle. In general, the differences in survival time and clinical duration between donor and recipients have to be interpreted with caution because only single animal to animal transmissions were done.

The presence of a positive scratch test in all atypical BSE-affected cattle was unexpected. Contrary to TSE-affected sheep where this sign is frequently observed [26,38] although the pathological mechanism that produces pruritus is unknown, it has only been reported in a small number of classical BSE cases [39,40] and is usually not tested in BSE-affected cattle. It is also not part of the routine examination protocol for signs of BSE in cattle used in this and other studies in our laboratory and was also not tested in the donor cattle because no response is usually elicited (T Konold, unpublished observation). A positive scratch response, however, can be observed in cattle suffering from *Chorioptes bovis* infestation (T Konold, unpublished observation), which often affects the skin around the tail head resulting in crusts and scabs (“tail mange”). We neither found skin lesions suggestive of Chorioptes mange nor did we observe signs of excessive pruritus on CCTV or weekly behavioural observations. Grooming behaviour was similar in atypical BSE-affected cattle and non-inoculated control cattle that were housed in the same pen but did not display a scratch response (data not shown). A larger sample size is required to evaluate whether this sign is specifically present in cattle with atypical BSE or an incidental finding, and it would be interesting to apply this test to recumbent cattle (downer cows) where no definite clinical diagnosis can be established and atypical BSE cannot be ruled out.

## Conclusions

Second passage of H-type and L-type BSE in cattle by intracerebral inoculation produced a TSE where the majority displayed the dull disease form regardless of disease form of the donor cattle. The pathological and molecular phenotypes of H- and L-type BSE were maintained.

## Additional files

Additional file 1:
**H5.** This steer shows difficulty rising on its hind limbs at 16.5 mpi. It sniffs the gloved hand of the clinician followed by head tossing and bobbing, nose licking and teeth grinding (‘BSE series of events’). The menace response and the response to touching of the head in the crush are considered normal. The steer is generally too reluctant to walk at a faster pace to appreciate gait abnormalities but hind limb ataxia is evident when it follows the other animal and crosses the hose on the floor. Scratching of the tail head elicits lip movements (positive scratch test). The steer can be seen standing in the pen and leaning its head against the bars of the pen division or resting it against the wall, with the eyes closed, which gradually slides down towards the floor. This episode lasts for approximately 8 minutes as indicated by the timer at the bottom of the clip. Additional file 2:
**L6.** This heifer is first seen walking along a corridor at 12.5 mpi, when its gait appears normal. It repeatedly responds to scratching of the tail head with lip movements and nose licking when examined 6 months later, which is not displayed by the control steer CO4 (inset), and has also developed hind limb ataxia. There is slight loss of balance after exiting the crush and it crosses its hind limbs occasionally when walking. The abnormal gait, which includes hind limb hypermetria, is more evident when the heifer trots. One day prior to cull, at 19.5 mpi, only slight difficulty getting up was noted on CCTV (rocking once before getting up; the heifer is indicated by a white arrow), but the heifer was found in lateral recumbency the next morning and only able to get up after being placed in a sternal position. When examined afterwards, there is mild over-reactivity to menace response testing and touching of the head. The scratch testing response is repeatable, more pronounced and can be elicited by scratching more cranial areas of the back, which results in head and neck movements followed by nose licking.
